# DROP app: A hydroclimate information service to deliver scientific rainfall, local rainfall, and soil moisture forecasts for agricultural decision-making

**DOI:** 10.1016/j.heliyon.2025.e42740

**Published:** 2025-02-18

**Authors:** Samuel Jonson Sutanto, Spyridon Paparrizos, Lisanne Nauta, Iwan Supit, Victoria Lefèvre, Gordana Kranjac-Berisavljevic, Bizoola Zinzoola Gandaa, Richard Dogbey, Baba Mohammadu Jamaldeen, Fulco Ludwig

**Affiliations:** a Earth Systems and Global Change Group, Wageningen University and Research, Droevendaalsesteeg 3, Wageningen, 6708 PB, the Netherlands; b Enabel, Development Agency of Belgium's Federal Government, 762 Kenneth Kaunda, Mozambique; c WACWISA, University for Development Studies, Mepukada, Tamale, Ghana

**Keywords:** Weather forecasts, Soil moisture forecasts, Forecast skills, Agriculture practices

## Abstract

Weather and Climate Information Services developed for agriculture often only provide scientific weather and climate forecasts on various timescales. Yet, local forecasts derived from indigenous knowledge and soil moisture information are still missing. In this study, we evaluate the implementation of the DROP app, a hydroclimate information service, offering both local (LF) and scientific rainfall forecasts (SF) and soil moisture forecasts, that was designed with and for smallholder farmers working on rainfed agriculture in northern Ghana. Results of the forecast assessment show that the LF generates a high probability of rain detection (POD), with a minimum value of 0.7. The hybrid forecast (HF) that integrates the SF and LF yields the highest POD value of 0.9 compared to others. However, the hybrid system also has a high number of false alarms which results in an overall lower forecast performance of HF compared to SF. Using forecasts obtained from the app, farmers adjusted their farming activities, such as time of sowing, planting and weeding dates, fertilizer and herbicide application, and harvesting. Although some limitations exist, the DROP app has potential to deliver actionable knowledge for climate-smart farm decision-making and thus, facilitate effective agriculture management.

## Highlights

• DROP app delivers scientific, local, and soil moisture forecasts to farmers.
• The scientific forecasts yield higher prediction skill than the local forecasts.
• Farmers are able to improve farming decisions, leading to improved yields.
• The app demonstrated its utility in facilitating effective agricultural management.

## 1 Introduction

Numerous Weather and Climate Information Services (WCIS) have been developed for different purposes [1], [2], [3], including those for agricultural decision-making [4], [5]. The WCIS for agriculture mainly provides scientific weather and climate forecasts on the timescale of days, weeks, or months, depending on the purposes of the services. Yet, the integration of farmers' indigenous knowledge to forecast weather i.e., local forecasts (LF) and information on soil moisture conditions are still missing. Many smallholder farmers in the Global South, especially in Africa, use local forecasts to predict rainfall events. This is partly due to the limitation of scientific forecasts (SF) in the provision of daily location-specific weather information [6]. Many farmers also believe that their LF based on the observed ecological indicators have a higher skill than the SF [7], [8].

Several studies recommend the development of a hybrid forecasting (HF) system that integrates SF with LF to improve forecast skill and increase acceptability among smallholders [9], [10], [11], [12]. These studies, however, do not provide guidance on how to develop the HF or report on its performance. Recent studies conducted by Gbangou et al. (2021) [13] and Nyadzi et al. (2022) [14] demonstrate that the HF has the potential to deliver skillful forecasts and can outperform any single forecast system. Additionally, the HF increases farmers' trust by incorporating their indigenous knowledge [15]. Farmers often believe that their forecasts based on observing local ecological indicators work better than the SF as this practice has been passed down from their ancestors to recent generations [16], [17].

Currently, most agricultural WCISs, focus on supply information on rainfall, especially from SF. However, soil moisture determines the plant growth and information on soil moisture is essential to optimize the agricultural yield [18], [19]. A study conducted by Sutanto et al. (2022) [20] shows that smallholder farmers in Ghana consider not only rainfall but also soil moisture conditions as important information for land preparation, sowing, fertilizer application, and harvesting. Born et al. (2021) [21] reported that soil moisture is important information to determine the planting dates. Moreover, soil moisture also provides crucial information on the intensity, duration, and timing of moisture stress under extreme events and mismanagement of agricultural practices [22]. For smallholder farmers, access to proper information on both rainfall and soil moisture can support their agricultural decision-making in mitigating the impacts of extreme events, reducing losses, rationalizing water use, and resulting in economic benefits, thus providing income security for many rural households [23], [24]. Therefore, developing a WCIS that integrates both scientific and local rainfall forecasts and is coupled with a soil moisture module is of utmost importance for smallholder farmers.

Recognizing the importance of location-specific, timely, and skillful information provided by climate information services, along with the need for integration of a soil moisture module, we have developed the DROP app, that is designed to address these requirements. The DROP app has features, which utilizes the dynamical weather forecasts from numerical weather prediction (NWP) models, incorporates the local weather forecasts derived from local ecological indicators from farmers, and uses a simple water balance model to forecast the soil moisture condition in the top soil layer. The objective of this article is to: 1) describe the features of the DROP app such as its ability to provide local rainfall and soil moisture forecasts, which are novel features compared to any farmer/weather app, 2) evaluate the accuracy (skill) of the scientific, local, and hybrid forecasts generated by the app, which combine both scientific and local forecast knowledge, and 3) study the extent to which the app can facilitate changes in farming activities, serving as an effective adaptation strategy for farmers.

## 2 Tool and methods

### 2.1 Implementation locations

**Figure 1:**
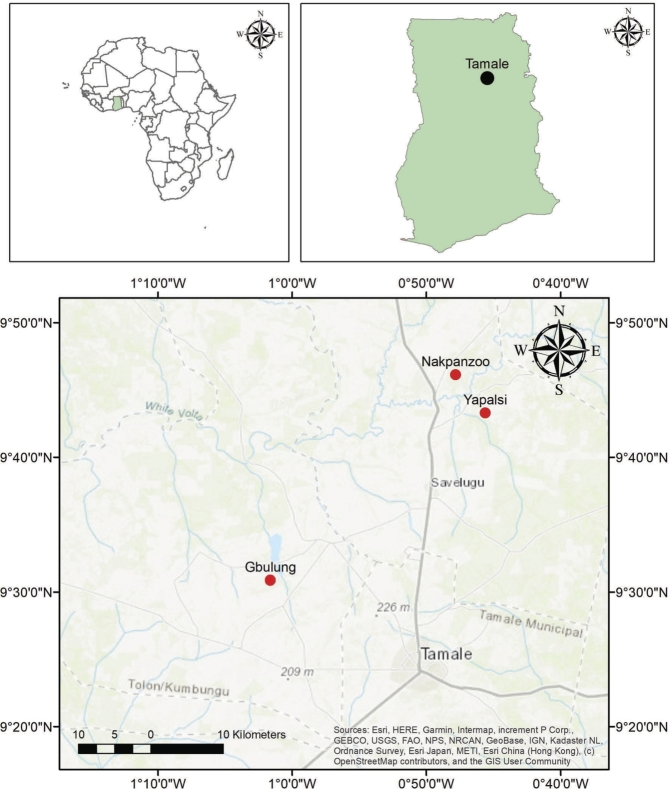
Map showing locations of the DROP app implementation area in northern Ghana. Gbulung is located in the Kumbungu district and Nakpanzoo and Yapalsi are located in the Savelugu district.

The testing and implementation of the DROP app were conducted in three communities, namely Gbulung in the Kumbungu district, northwest of Tamale, and Nakpanzoo and Yapalsi in the Savelugu district north of Tamale, the regional capital (Fig. 1). These districts are located in the Northern Region of Ghana, that has the highest rice production across the country, and many smallholder farmers engage in rainfed agriculture. We have tested and evaluated the DROP app in the same communities as the baseline study in order to avoid conducting basic training on weather forecasting again [25], [5]. Moreover, farmers in these three communities are familiar with the use of the mobile app to acquire weather forecasts e.g., via the Farmer Support app [25], [5]. Rainfall data, including observations and forecasts, have been collected in these communities since 2020 using the Farmer Support app, the predecessor of DROP app. Geographically, the farmers' communities are located between latitudes 930′∘0″ - 948′∘0″ North and longitudes 102′∘0″ - 045′∘0″ West, and at an elevation of about 160 m a.s.l. The region experiences six months rainy season from May to October and six months of dry season from November to April, with total rainfall below 1,250 mm [26], [27]. The soil type in the study areas is mostly sandy loam with alluvial deposits found in the lowland regions. The DROP app field implementation was done in the period June-August 2022 and monitoring and feedback were performed from September to October 2022. Both the testing and evaluation periods were carried out during the rainy season.

### 2.2 The DROP app and its main features

The DROP app is a research-based, tailor-made hydro-climate information service that is co-produced with and for farmers in Ghana (Fig. 1). The co-production process involved scientists, app developers, and farmers to identify their information needs and requirements. The co-production processes actively engaged farmers to harness their knowledge and experience in order to develop WCIS that is tailored to smallholder context-specific needs [28] and in the end increase trust and chances of information uptake [29]. Focus group discussions (FGDs), farmers' training, interviews, and questionnaires (using Kobotoolbox, www.kobotoolbox.org) were performed to gather information on the needs of information for agricultural activities. In each community, with the help of local contact persons, around 20 farmers (total 62) have been selected to participate in the interview and questionnaire. In each group, consisting of 10 male and 10 female farmers, we had an average of four people who were speaking English. During the FGDs and training in three communities (Fig. 1), we trained the farmers on how to use the scientific forecasts, how to fill in data from the local forecasts and observation, how to provide the soil moisture initial condition, and interpret the soil moisture forecasts. Some tablets were also provided for farmers who do not own smartphones. Monitoring and evaluation of the app were conducted after it was used by the farmers for a minimum of one month. Farmers also provided feedback for future improvements of the app.

The DROP app offers scientific rainfall forecasts with 1- to 14-day lead times, local rainfall with 2-day lead time, and 7-day soil moisture forecasts. Farmers can select the crop type and the planting date for their fields. Farmers can also add additional crops in the same field and edit their planting dates. The currently available crop types are Rice, Maize, Cowpea, Groundnut, and Leafy Vegetables, which are the most common crops in northern Ghana. Share forecast is an option where farmers can share their own forecast based on local indicators they observed. We equipped farmers in our study regions with simple rain gauges, as no Ghana Met Office (GMET) rain gauges are installed nearby. Farmers measure the precipitation every morning at 9:00 AM local time and fill in the rainfall observation taken from local measurements. This feature also served as a database for local rainfall that is used to evaluate the performance of the SF and LF forecasts. The next feature is viewing the forecasts where farmers can see both the LF issued by their peers in the same location and SF provided by global weather models. The last feature is the soil moisture information section. In this section, farmers can fill in the soil moisture initial condition by measuring the soil moisture condition or using data taken from the low-cost sensors, which is needed for the calculation of soil moisture forecasts, and irrigation planning for 7 days ahead. After farmers defined the soil moisture initial condition and irrigation planning, the soil moisture forecast is displayed.

### 2.3 Rainfall forecasts

#### 2.3.1 Local rainfall forecasts

**Figure 2:**
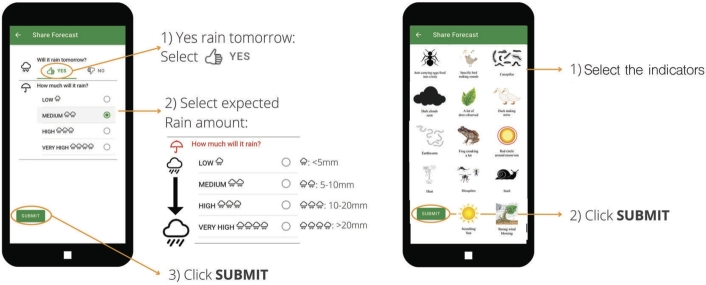
a) Farmers' local prediction for rainfall (low, medium, high, and very high) and b) 14 local indicators that farmers observed to predict the rainfall.

Many studies show that smallholder farmers around the world use local knowledge to predict the weather [16], [30], [17], [31]. This is also the case for smallholder farmers in Ghana [13], [32]. Gbangou et al. (2021) [13] described 22 indicators used by farmers in the greater Accra region of Ghana to predict the weather. These indicators can be divided into five categories, which are atmospheric (clouds, wind, dew), astronomic (sun, sky, moon), fauna (ants, frogs, birds, goats, scorpions, worms, pigs), and flora (trees). From these 22 indicators, only 14 indicators were selected to be used in the DROP app because farmers in Ghana mostly use these indicators to predict the weather. These indicators are ants, bird, caterpillar, dark cloud, dews, duck, earthworm, frog, red circle around moon/sun, heat, mosquitos, snail, scorching sun, and strong wind (Fig. 2b). The local forecast (LF) feature embedded in the DROP app provides an opportunity for farmers to submit their own local forecasts and share their forecasts among peers.

The local rainfall forecasts are provided for today's forecast (a lead time of 1 day according to SF) or tomorrow (a lead time of 2 days). Today's forecast must be filled in before 9.00 AM in the morning local time for rainfall prediction from 9.00 AM until 11.59 PM on the same day. Tomorrow's forecast can be submitted before 12.00 AM today (midnight) to predict the rainfall event the day after. Farmers provide their perception of the precipitation (P) amount, such as low (P<5 mm), medium (5≤P<10 mm), high (10≤P<20 mm), and very high (P≥20 mm) (Fig. 2a). However, it is difficult to estimate the rainfall amount based on local predictions since the appearance of ecological indicators only predicts rain or no rain occurrences. We also did not categorize which indicators correspond to low, medium, high, and very high rainfall amounts. Fig. 2b shows the local indicators embedded in the DROP app. The selection of symbols was consulted with the local farmers in our farming communities. In the DROP app, farmers can select multiple indicators.

#### 2.3.2 Scientific rainfall forecasts

Currently, the DROP app utilizes the SF provided by a weather provider (i.e., meteoblue). The forecast data are fetched to the app through an API:URL, which delivers the forecasts for today, 7 days, and 14 days in advance. meteoblue utilizes the NOAA Environmental Modelling System (NEMS [33]) for a worldwide high-quality forecast. The model has diverse horizontal resolutions of 4 km to 30 km and a vertical resolution of 100 m to 2 km, depending on the region of the world. The forecasts are updated twice a day at 12.00 AM (midnight) and 12.00 PM UTC (noon). The displayed information in the DROP app is rainfall probability (%) and rainfall categories, such as low (P<5 mm), medium (5≤P<10 mm), high (10≤P<20 mm), and very high (P≥20 mm) in different colors.

#### 2.3.3 Hybrid forecasts

While LF forecasts rely on rainfall predictions derived from local indicators observed by farmers, and SF is based on numerical weather model, the hybrid forecast (HF) is developed by integrating both local and scientific forecasts. An integration between SF and LF may offer a skillful forecasting system as it was acknowledged by some studies [34], [17]. These studies, however, did not attempt to develop the integrated system, hereafter namely the HF. To the authors' knowledge, there are only two studies that combined the SF and LF and evaluated the forecast skills of the HF [13], [14]. In this study, we have tried to develop a simple HF system based on the occurrences of rainfall events that were detected by one of the forecast systems, either SF or LF. We found during the FGDs that the local indicators used to predict rain and no rain events were not always visible. Therefore, farmers solely depend on the SF. In this system, rain is predicted if any of the forecasts show its possible occurrence. No rain event is predicted if both forecasts do not predict rain (Table 1). This system differs from previous studies that employed the statistical approach to integrate the SF and LF based on the LF probability [14] and the number of indicators observed [13]. Our study is limited by the number of indicators observed by farmers and the number of farmers who filled in their LF. Therefore, we offer another approach that is simple to be integrated into the system and based on the type of LF data we have [35]. Rain is predicted if more than 50% of farmers forecast rain based on LF or if the probability of SF predicting rain exceeds 50%. If either condition is met, then the HF predicts rain.

**Table 1:** The prediction of rain and no rain events for the simple Hybrid Forecasts (HF). SF stands for scientific forecasts and LF stands for local forecasts.

Forecasts		
SF	LF	HF
Rain	No rain	Rain
No rain	Rain	Rain
Rain	Rain	Rain
No rain	No rain	No rain

### 2.4 Soil moisture forecasts

The soil moisture module is developed based on both scientific and local approaches. The scientific approach includes a soil water balance model, meteorological input, crop parameters, and soil characteristics, while the local approach includes farmers' cropping calendar and estimation of soil moisture initial condition measured by farmers. Soil types for each farmer's location are automatically determined from the DROP app's soil database, based on the coordinates of farmer community locations when they register their fields. The DROP app utilizes the global soil type data from the ISRIC world soil information database [36]. The detailed soil moisture water balance method is provided in the Supplementary Information. The training on soil moisture measurements was conducted in three farming communities. Soil moisture measurements are required to obtain the soil moisture initial conditions for the soil water balance calculation. The soil moisture initial condition is estimated using three methods. The first is an on-site measurement by farmers, the second is from the remote sensing dataset (i.e., Vandersat, now the Planet), and the third is using the previously forecasted soil moisture. The first approach, feel and appearance method introduced by the US Department of Agriculture (USDA), has a measurement accuracy of 5% for experienced observers [37]. The Vandersat remote sensing data has accuracy of approximately 0.05 m^3^/m^3^, depending on the vegetation cover [38]. The last approach, which is based on the previously forecasted soil moisture as the current day's initial condition is less favorable to be used. These three approaches are being employed in the DROP app and farmers can decide which method they want to use. If the farmers do not provide their observations and no data is fetched to the system through the API:URL, then the drop app will use the soil moisture forecasted from the previous day as the current day's soil moisture initial condition (third approach).

During the baseline study (see Sutanto et al. (2022) [20]), our team trained the farmers on how to estimate soil moisture on the field, using the feel and appearance method [37]. The feel and appearance of soil vary with soil type and moisture content. This method can be used to obtain the soil moisture initial condition for the DROP app to estimate the soil moisture forecasts. The percentage of soil moisture needs to be entered into the app by sliding the soil moisture color bar. We also equipped farmers with low-cost soil moisture sensors (Xiaomi Flower Care Plant Sensor). The data measured from these sensors can be transferred to the farmers' mobile phones. This sensor can be used by technologically more advanced farmers, usually from the younger generation, to estimate the soil moisture initial condition, instead of using the feel and appearance method.

### 2.5 Forecast skill evaluation

A dichotomous forecast method is employed in our study to verify forecasted events that will occur (“yes”) and will not occur (“no”) [39], [40]. This method is built by developing a contingency table that shows the frequency of “yes” and “no” forecasts and observations (Table 2). Rain was observed or forecasted if rain with a value of >0.1 mm is observed or forecasted. A hit is counted when both the forecast and observed show a rainfall event and a miss is counted when the forecast shows no rainfall event, but it did occur. A false alarm is counted when the forecast indicates a rainfall event, but it did not occur. Lastly, the correct negative is counted when the forecast predicts no rainfall event, and it did not occur. The probability of detection (POD), the false alarm ratio (FAR), and the Hanssen-Kuipers discriminant (HK) metrics are statistical metrics commonly used to determine the accuracy of forecasts [41]. POD, FAR, and HK have scores ranging from 0 to 1, with a skillful prediction if the POD and HK values are close to 1. FAR, on the other hand, indicates a skillful forecast if the FAR value is close to 0. All the skill metrics used in this study have been widely applied to verify the skill of climate predictions [42], [13], [43], [44].

**Table 2:** Contingency table that shows possible combinations of forecasted and observed rain events.

		Event observed		Total
		Yes	No	
Event forecasted	Yes	Hits	False alarms	Forecast yes
	no	misses	correct negatives	forecast no
Total		observed yes	observed no	total

The POD, FAR, and HK are calculated as follows (see also Table 2):

(1) $$POD=\frac{hits}{hits-misses}$$

(2) $$FAR=\frac{falsealarms}{hits+falsealarms}$$

(3) $$HK=\frac{hits}{hits-misses}-\frac{falsealarms}{falsealarms-correctnegatives}$$

For the forecast skill evaluation, we only used the combined data from Nanpakzoo and Yapalsi. The Nanpakzoo community is located close to Yapalsi (<10 km, Fig. 1) thus the SF forecasts (not hindcast) for Nanpakzoo and Yapalsi are the same. These locations were also used in the previous study [5], where the authors collected the LF data during the rainy season of 2020. In the current study, we extended the data collected from the previous study and data obtained during the DROP app implementation. Data collected from Gbullung was not used because we only have LF data collected during the DROP app implementation. Therefore, the LF data for Gbullung is too short for robust analysis. In addition, there was no rain gauge installed in Gbullung before the DROP implementation. In total, we observed 47 rainy days, with SF predicting rain on 61 days and LF predicting rain on 53 days.

## 3 Results and discussions

### 3.1 Rainfall and soil moisture forecasts

**Figure 3:**
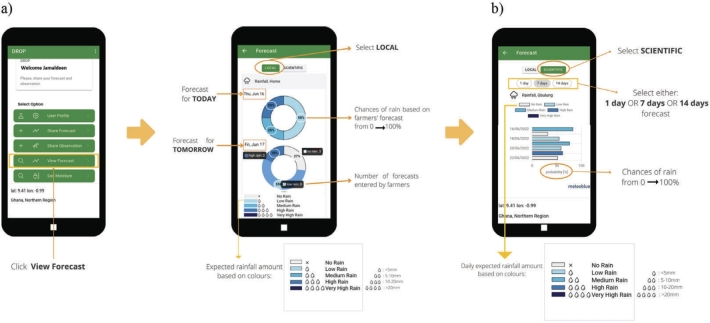
a) Example of the local precipitation forecast provided by the farmer and b) scientific weather forecasts for 1-day, 7-day, and 14-day retrieved from the meteoblue API:URL by the DROP app.

Fig. 3a shows the precipitation forecast for the current (lead time of 1 day) and the next day (lead time of 2 days) derived from local indicators (see Fig. 2b for indicator types). The forecasts are presented as pie charts. From the first pie chart, half of the participants (6 farmers from a total of 12 gave predictions) predicted that low rain would occur on Thursday 16 June 2022 and 3 farmers each predicted medium and high rain. Farmers observed ants and wind as indicators to predict the rainfall event on 16 June 2022. In the app, we use the number of farmers submitting their forecasts to determine the rainfall probability in the local forecast. For the next day's weather (17 June 2022), 27% of farmers (3 farmers) predicted that no rain would occur and only a few numbers of farmers (18% out of 11 farmers) predicted a high rain event. The majority of the farmers (55%) observed medium rain. The observed indicators were heat, a red circle around the moon, and mosquitos. We noted that 12 farmers submitted their forecasts for 16 June 2022 but only 11 farmers submitted their forecasts for 17 June 2022. This study does not evaluate which indicators perform best in forecasting rainfall. However, Nyadzi et al. (2022) [32] identified butterflies, bird, mosquitoes, and caterpillars as the most reliable indicators for rainfall prediction in northern Ghana. Interestingly, farmers in our study areas did not select butterflies as a local indicator for rainfall forecasting, so this indicator was excluded from the DROP app.

Fig. 3b shows the panel where the farmers can obtain the forecasts and the 7-day scientific forecasts of rainfall (meteoblue forecast). For example, based on the 7-day forecast, medium rain was expected to occur in the Gbulung farming community on 16 June 2022 and 19 June 2022, with a probability of 80% and 75%, respectively. These probabilities are derived from the frequency of precipitation occurrences across an ensemble of 20 different forecast runs. For rain events, we classify rain events based on the amount of rainfall more than 0.1 mm. Thus, we neglect the influence of very light drizzle in a day with a value of less than 0.1 mm, which is in agreement with previous studies [45], [44]. The occurrence of light drizzle is depicted in Fig. 3b where a no rain event was forecasted with a probability of 30% on 17 June and 22 June 2022 (gray bars). By definition, the probability of no rain means a rainfall amount of less than 0.1 mm.

The decision on the forecast lead times (1-, 7-, and 14-day) was taken after the baseline study was conducted [20]. Farmers in the study areas do not consider information with longer lead times as important as short lead time forecasts. This differs from previous studies in northern Ghana, where one month lead time was chosen by many small-scale rice farmers with irrigated farms [46], [47]. Another study by Kumar et al. (2020) [48] shows that smallholder farmers in Bangladesh requested weather forecasts with lead times of 7-day and 14-day.

**Figure 4:**
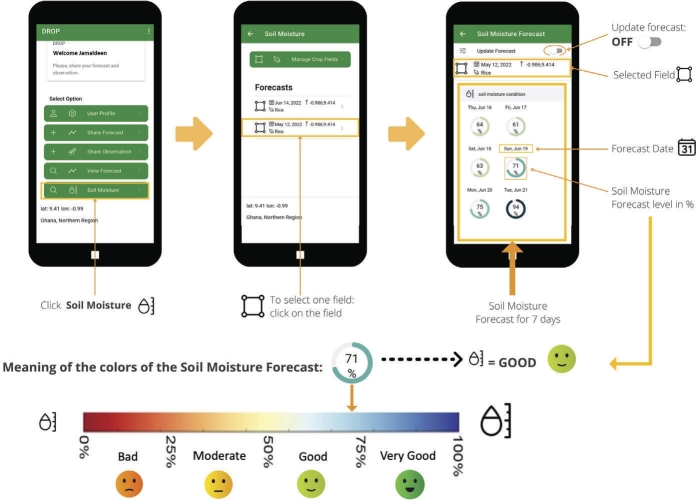
Soil moisture forecast for 16 June 2022 with 7 days lead time.

Fig. 4 shows the forecasted soil moisture from 16 June 2022 to 21 June 2022 following the rainfall pattern. For example, the predicted soil moisture condition on 16 June 2022 is relatively wet (64%) due to the forecasted rainfall event (see Fig. 3a). A significant increase of soil moisture from 75% to 94%, almost fully saturated, is seen on 21 June 2022, when a high rainfall event was forecasted. We used the color bar plus symbols as depicted in Fig. 4 to indicate the meaning of soil moisture in the easiest way for illiterate farmers. Blue color means that soil moisture condition is good (wet) and very good (very wet) and vice versa for red color. During the testing period, only around 5 farmers in each community provided the soil moisture initial condition data. The number of farmers filled in the data is lower than we expected, which could be caused by the complexity of how to provide soil moisture initial condition to the app. This calls for simplification of the soil moisture module or more training on soil moisture forecasts.

### 3.2 Skill of rainfall forecasts

**Figure 5:**
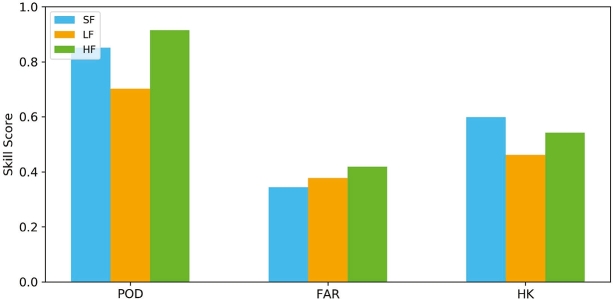
The performance of scientific forecast (SF), local forecast (LF), and hybrid forecast (HF). POD stands for probability of detection, FAR stands for false alarm ratio (FAR), and HK stands for the Hanssen-Kuipers discriminant.

In this study, we evaluated the performance of SF, LF, and HF forecasts that was derived from a simple integration approach based on rainfall prediction. Assessment of forecast skills was carried out for combined data from Nanpakzoo and Yapalsi. These locations are nearby and have long records of LF data, compiled from Paparrizos et al. (2023) [5] and this study. The farmer community in Gbulung was not selected because the collected data is too short for analysis. Fig. 5 shows that the SF has a higher probability of detection rate (POD) compared to LF, with a POD value of 0.85. This means that 85% of rain events (rain>0.1 mm) were predicted by the SF. The LF yields a lower POD of 0.7 but higher FAR value. Moreover, the high POD obtained from SF is supported by the low false alarm (FAR) value of SF compared to LF (FAR=0.34 and 0.37, respectively). A high false alarm means that the forecast predicted rainfall that did not occur, which may reduce the users' trust in the credibility and consistency of the forecast [49], [50]. High POD and low FAR cause the overall performance of SF higher than the LF, denoted by the Hanssen-Kuipers discriminant (HK) values of 0.59 and 0.46, respectively.

The DROP app utilizes the meteoblue forecast derived from the NEMS model at a 30 km spatial resolution for Africa. However, the accuracy of the model in simulating precipitation at the local scale is inherently limited. This limitation arises from challenges in cloud parameterization for simulating convective precipitation and the methodologies used for ensemble perturbation [51]. Such constraints are common across all numerical weather prediction models, including NEMS. For improved performance at the local scale, the use of regional climate models (RCMs) is recommended. RCMs typically offer finer spatial resolution and are better equipped to capture localized weather phenomena, thus providing more reliable precipitation forecasts tailored to specific areas.

The Hybrid Forecast (HF), which is an integration of SF and LF, indeed results in a higher POD score than SF and LF (POD=0.91). However, HF also generates a high FAR value of 0.42, which is the highest compared to others. Our results show that both LF and HF yield high FAR, associated with over prediction of rainfall event. Missing interpretation of the occurrences of local indicators by inexperienced farmers may lead to inaccurate forecasts. Additionally, HF's reliance on rainfall prediction amplifies the likelihood of forecasting rain events that do not occur [35]. Overall, the HF outperforms LF (HK=0.54) but it has a slightly lower performance than SF. The high HK values (HK>0.35) obtained from all the forecasting systems indicate that the forecasts can discriminate between yes/no events [41], [13].

Previous studies in assessing HF performance concluded that HF generates higher performance than SF and LF [13], [14], which is not the case for our study regions. The simple integration method based on the precipitation occurrences (see Section 2.3.3) yields high FAR although it generates high POD. Nevertheless, the performance of HF is higher than LF, which has been used by farmers in Ghana for generations. Developing the HF is, therefore, recommended because the farmers' indigenous knowledge is included in the system, which may increase the trust among farmers regarding this new technology and result in better uptake of forecast information. In addition, providing one single integrated forecast will minimize the confusion among farmers when the forecasts are contradictory and which forecast should be chosen [14]. A more sophisticated method than a simple approach implemented in this study might contribute to skillful HF. Currently, we are developing a machine learning algorithm to combine scientific and local weather forecasts. When completed, the algorithm will be encapsulated in the DROP app to generate skillful HF forecasts. In the future, the DROP app will offer all three forecasting systems, allowing farmers to select the forecast type they prefer to use. Some farmers, particularly older generations, may continue to trust their local knowledge (LF), while younger generations may favor modern technology (SF) and ideally HF.

### 3.3 Changes in farming activities

**Figure 6:**
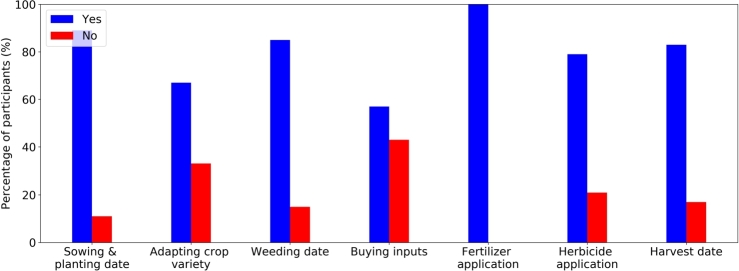
Changes made by farmers (n=27) based on forecast information obtained from the DROP app.

Result obtained from a questionnaire conducted at the end of the implementation period shows that farmers have adjusted their farming activities based on the forecasted rainfall and soil moisture. Fig. 6 shows the changes in farming activities after the implementation of the DROP app. The main agricultural adaptation activities were a modification of the sowing and planting, as well as weeding date, fertilizer and herbicide application, and harvesting date. Most farmers (89%) reported that they changed their sowing schedules based on the soil moisture conditions. By doing this, they can sow when soil moisture condition is more optimal, possibly increasing their yields and preventing re-sowing. The changes in the weeding date were reported by 85% of the respondents. Farmers stated that wet soil is not recommendable for weeding and thus, the prediction of high or low soil moisture helps them to determine the weeding timing. All respondents also claimed that they consult the precipitation forecasts obtained from the DROP app to indicate the fertilizer timing. Fertilizer will not be applied if the DROP app predicts rain occurrence on that day. The rain will wash away the fertilizer, which could lead to reduced fertilizer availability in the soil. Reapplying fertilizer increases the amount of fertilizer used and could potentially increase environmental pollution. The same is also applicable to herbicide applications. 83% of the farmers also changed their schedule for harvesting their groundnuts, soya beans, and maize based on the prediction of soil moisture and rainfall. Harvesting groundnuts is best to be done when the soil is not too dry, according to some farmers. Farmers also stated that they will harvest the soya beans or maize when no rain is predicted. In the end, the app allows farmers in the studied regions to better organize the harvest and drying schedules.

Detailed information on the changes in farming activities, after farmers use the app, was obtained by interviewing 27 farmers. We note that we only interviewed farmers who filled in the soil moisture questionnaire. For instance, farmers explained that the onset of the rainy season indicates the start of the sowing of maize. In April 2022, after the first rainfall event, farmers started sowing maize. However, the second rain occurrence came too late which prevented the seeds from germination and the crop had to be resown, resulting in the loss of money. The forecasts obtained from the DROP app provide opportunities to prevent this and can have a direct impact on the income and expenditures of the farmers who do not need to buy new seeds or use some of their food provisions. A few farmers also stated that the forecasts improve their livelihood by adjusting the fertilizer timing. According to them, the increase in fuel prices has an impact on the cost of fertilizers in 2022, which is more than double compared to the price in 2021. The considerable increase in fertilizer prices results in much higher costs for farmers for growing crops. Farmers explained that the DROP app allowed them to optimize the economic value of fertilizer use by applying it during suitable environmental conditions and reducing waste due to leaching. During the harvesting season of the groundnuts in August, female farmers explained that the soil moisture forecasts allow them to determine the best days for harvesting. The soil should not be too dry or too wet to be able to harvest the groundnuts. Furthermore, once the nuts are harvested, farmers need to dry them in the sun for three to five days. During this time, a weekly rainfall forecast is the key to avoid groundnut wetting, leading to the germination of the nuts.

### 3.4 Limitation of the DROP app

Despite positive feedback obtained from the farmers on the use of the tool, some relevant limitations arose during the app implementation and evaluation. These would need to be addressed in the future to further foster DROP app implementation in small scale agricultural communities to truly benefit numerous farmers. One of the key issues is limited network coverage in the study areas and the limited number of people who own smartphones. Therefore, an offline mode is now being implemented where farmers can open the app, input the data, and latter get the updated information when the internet network is available. In addition, users have issues understanding all the features of the DROP app. The app was reported as easy to use by the majority of the participants. However, monitoring revealed that some users still rely on other participants to have access to forecast information. Hence, future app implementation should include a more extensive training component. Another barrier is the English language used in the DROP app, which affects users with limited English skills. To address this, future app versions could be implemented in local languages.

Entering the initial soil moisture is complicated for the majority of farmers. According to them, this was due to the lack of clearness of the soil moisture forecasts and the necessity to report moisture estimation in percentages. Farmers who did not use the soil moisture forecast explained that they do not understand the objective of the soil moisture forecast. They prefer to look directly in the field for the soil moisture initial condition based on their local knowledge to take a farming decision. A few farmers understand the usefulness of knowing the soil moisture condition but not the potential advantage of the forecasts. Farmers who understood the need for soil moisture forecast knowledge managed to plan their activities based on the forecasts as discussed in the previous section.

## 4 Conclusions and recommendation

The proof-of-concept of the DROP app has been applied in the field by promoting a tailored service to meet the farmer's needs and to build the trust of the farmers, through the co-production process. In this paper, we illustrate some key novel features of the app, such as providing scientific rainfall, local rainfall, and soil moisture forecasts, evaluate the forecast performance, and describe how the use of the app changes agriculture decision-making. The app is able to forecast rainfall events derived from SF and LF, leading to an increase in soil moisture. Our study shows that SF outperforms the LF in our study regions. The integrated forecast i.e., hybrid forecast (HF) performs better than both SF and LF in rainfall detection (high POD). Overall, the HF yields better forecast performance than LF, indicating that developing the HF is of utmost importance to generate a trustworthy and skillful forecasting system for smallholders. By using the rainfall and soil moisture information provided by the app, farmers are able to improve farming decisions regarding the time of sowing, planting and weeding, fertilizer and herbicide application, and harvesting. This can potentially improve yields and the economic performance of farming activities.

The DROP app, a research-developed tool, has the potential to be implemented in agricultural economies of the global, as its SF data is accessible worldwide. However, the use of LF depends on the availability of region-specific local indicators. While the app has integrated local indicators commonly used in Ghana, farmers in other regions might rely on different local indicators for rainfall prediction as highlighted by Paparrizos et al. (2023) [52], who identified over 1300 such indicators. This indicates that customization of the LF feature will be required when deploying the app in different regions. Furthermore, the crop type database within the app currently includes crops common to Ghana, Guatemala, and Bangladesh [53]. For the app to be used effectively in other regions, this database needs to be expanded to include relevant crops and their development stages for those regions. To conclude, while the DROP app can be implemented globally, its successful implementation in new locations requires updates and adjustments, particularly in terms of local indicators and crop data. The use of the app as showed in this study has the prospective to allow farmers to adapt farming practices using climate and weather information improving the agriculture potential in a world faced with climate change.

## CRediT authorship contribution statement

**Samuel Jonson Sutanto:** Writing – review & editing, Writing – original draft, Validation, Supervision, Methodology, Investigation, Formal analysis, Conceptualization. **Spyridon Paparrizos:** Writing – review & editing, Supervision, Project administration, Investigation, Funding acquisition, Conceptualization. **Lisanne Nauta:** Visualization, Validation, Software, Data curation, Conceptualization. **Iwan Supit:** Writing – review & editing, Supervision, Resources, Methodology, Conceptualization. **Victoria Lefèvre:** Visualization, Investigation, Formal analysis, Data curation. **Gordana Kranjac-Berisavljevic:** Writing – review & editing, Supervision, Resources. **Bizoola Zinzoola Gandaa:** Supervision, Resources, Data curation. **Richard Dogbey:** Resources, Formal analysis, Data curation. **Baba Mohammadu Jamaldeen:** Resources, Investigation, Data curation. **Fulco Ludwig:** Writing – review & editing, Supervision, Project administration, Funding acquisition.

## Declaration of Competing Interest

The authors declare the following financial interests/personal relationships which may be considered as potential competing interests:

Spyridon Paparrizos reports financial support was provided by the ERA-NET-COFUND. If there are other authors, they declare that they have no known competing financial interests or personal relationships that could have appeared to influence the work reported in this paper.

## Data Availability

Data will be made available on request.

## Appendix A Supplementary material

MMC Supplementary Material “DROP app: a hydroclimatic information service to deliver scientific rainfall, local rainfall, and soil moisture forecasts to smallholder farmers”. In the supplementary material, we provide a detailed concept of soil moisture water balance, soil parameters, and evapotranspiration calculation. MMC
