# Supplementary material for: DROP app: A hydroclimate information service to deliver scientific rainfall, local rainfall, and soil moisture forecasts for agricultural decision-making
Source: Heliyon. 2025 Feb 18;11(4):e42740. doi: 10.1016/j.heliyon.2025.e42740 (PMC11883362; doi:10.1016/j.heliyon.2025.e42740)
Supplement: MMC — Supplementary Material “DROP app: a hydroclimatic information service to deliver scientific rainfall, local rainfall, and soil moisture forecasts to smallholder farmers”. In the supplementary material, we provide a detailed concept of soil moisture water balance, soil parameters, and evapotranspiration calculation. [file mmc1.pdf]

SUPPLEMENTARY MATERIAL

DROP app: a hydroclimatic information service to deliver scientific rainfall, local rainfall, and soil moisture forecasts to smallholder farmers

Samuel J. Sutanto[1], Spyridon Paparrizos[1], Lisanne Nauta[1], Iwan Supit[1], Victoria Lefèvre[1,2], Gordana Kranjac-Berisavljevic[3], Bizoola Z. Gandaa[3], Richard K. Dogbey[3], Baba M. Jamaldeen[3], Fulco Ludwig[1]

[1] Water Systems and Global Change Group, Wageningen University and Research, P.O. Box 47, 6700 AA, Wageningen, The Netherlands

[2] Enable, Development Agency of Belgium's Federal Government, 762 Kenneth Kaunda, Mozambique

[3] West African Centre for Water, Irrigation and Sustainable Agriculture (WACWISA), University for Development Studies, Tamale, Ghana

CORRESPONDING AUTHOR: Samuel Jonson Sutanto, email: samuel.sutanto@wur.nl.

In the supplementary material, we provide a detailed concept of soil moisture water balance, soil parameters, and evapotranspiration calculation.

SOIL MOISTURE METHODS

Soil moisture water balance

A simple conceptual model of soil water balance was used to simulate soil moisture content in the active root zone (Rao, 1987). The model used includes physical processes like infiltration from rainfall or irrigation, and plant water uptake in the form of evapotranspiration. The DROP app utilizes the one layer of soil water balance model. One layer here is the active root layer or top layer where both moisture extraction by evapotranspiration and percolation would occur. Hereby, the soil water flow is only described in the vertical direction. This model does not require too many or complicated parameters and can be suitably used to monitor soil water content in the root zone of any crop (Panigrahi and Panda, 2003). The model runs with a constant time step of twice a day following the forecast initiation times. The soil water balance, in principle, simulates the changes of water in the soil reservoir (ΔSM) and is estimated by the difference in input water (In) and output (Out):

$$\Delta SM = In - Out \tag{1}$$

The input components consist of precipitation (P) and irrigation (IR) and the output is evapotranspiration (ET). Since we apply only one layer model, the percolation water to the deeper layer (second layer) is neglected in the water balance calculation. The change in soil moisture is derived from the difference between soil moisture at time t and at time t-1. Thus equation 1 can be derived as:

$$SM_t - SM_{t-1} = P_t + IR_t - ET_t \quad 2)$$
$$SM_t = P_t - ET_t + SM_{t-1} + IR_t \quad 3)$$

with SM_t is the soil moisture condition at time t (forecast), P_t is the rainfall at time t (forecast), ET_t is the evapotranspiration at time t (forecast), SM_{t-1} is the soil moisture condition at time t-1 (initial condition), IR_t is irrigation water at time t (planning) if it is applied, and t itself is the forecast lead times, which are 1-day and 7-day for a weekly forecast. For rainfed agriculture without irrigation, the IR_t is zero all the time. In the DROP app, irrigation water needs to be provided by farmer by choosing medium (50% from saturation) or full irrigation (100% or saturated). These two simple options were chosen because farmers never measure the irrigation water. Farmers living close to the river just simply add water from the river to the field until its wet (50%) or water pounding is occurred (100%).

We need to consider the infiltration capacity in our calculation (I). The input components, such as combined P_t and IR_t, should not exceed the infiltration capacity, I (Equation 4). If this is the case, then the excess water will be treated as runoff.

$P_t + IR_t - ET_t < I$, see Table S1 for infiltration values (I) 4)

Soil parameters

The global soil type is taken from the ISRIC World Soil Information database (Batjes, 2009). The ISRIC soil database consists of 13 soil types, which are clay (heavy), silt clay, clay (light), silt clay loam, sandy clay, clay loam, silt, loam, silt loam, sandy loam, sandy clay loam, sand, and loamy sandy. The soil profile may be composed of several soil layers, each with their specific characteristics, e.g., the infiltration rate, wilting point, field capacity, and saturation. For simplicity, we grouped the soil types into five main soil classes, which are clay, clay loam, loam, sandy loam, and sand. Each soil type has different infiltration rates, starting from the fastest (sandy soil) to the lowest (clay soil). Table S1 presents the basic infiltration rate for different soil types (FAO, 1988).

Table S1. Basic infiltration rate (I) for various soil types

Soil type	Basic infiltration rate (mm/hour)
Sand	< 30
Sandy loam	20 – 30
Loam	10 – 20
Clay loam	5 – 10
Clay	1 – 5

Other soil parameters that are important in the calculation of soil water balance are soil field capacity and wilting point. One should be noted that the forecasted soil moisture content (SM_t) must not be higher and lower than the field capacity and wilting point, respectively. The field capacity and wilting point of each soil type are calculated using the Pedotransfer formula (Saxton et al., 1986) by considering different soil types that are found in the farmers' fields (script transferred to python).

The Pedotransfer script simulates the values of wilting point, field capacity, saturation, and plant available water for different soil types at any given location.

Evapotranspiration (ET) calculation

The potential evapotranspiration (PET_c) is a function of crop type, its growing stage, and climate under normal conditions. This means that no limitations are placed on crop growth or evapotranspiration from soil water and salinity stress, crop density, pests, and diseases, weed infestation, or low fertility. PET_c is determined by the crop coefficient approach where the effect of the various weather conditions is incorporated into reference evapotranspiration (ET_o) and the crop characteristics into the K_c coefficient:

$$PET_c = K_c\,.ET_o \qquad 5)$$

There are many methods to estimate the ET_o, with different input data requirements. In the DROP app, the ET_o is calculated using the Hamon method, which requires only temperature and daytime length (Lu et al., 2005; Paparrizos et al., 2017). The ET_o (mm d^{-1}) is calculated as follows:

$$ET_o = 0.1651\,.L_d\,.RHOSAT\,.KPEC \qquad 6)$$

where L_d is the daytime length, which is the time from sunrise to sunset (hours), RHOSAT is the saturated vapor density (g m^{-3}) at the daily mean air temperature (T) in °C, $KPEC$ is the calibration coefficient, which is set to 1.2 in this study. $RHOSAT$ is calculated as follows:

$$RHOSAT = 216.7\,.ESAT/(T + 273.3) \qquad 7)$$
$$ESAT = 6.108\,.EXP(17.26939\,.T/(T + 237.3)) \qquad 8)$$

where $ESAT$ is the saturated vapor pressure (mb) at the given T.

The values of K_c differ for every crop and for every crop development stage, such as initial stage, development stage, mid stage, and late stage. At the beginning of the app, we ask the farmers to fill in what crop they will plant and when they will start planting. By doing this, farmers are engaged in the identification of crop types and cropping calendar information over the study location. FAO (1998) provides general lengths for the four distinct growth stages and the total growing period for various types of climates and locations. Here, we will provide the lengths of crop development stages for the Ghana region and for specific crops that are commonly planted by farmers. Table S2 provides information on K_c values and the crop development lengths for different crops in Ghana.

Table S2. K_c values and the lengths of crop development stages in day for Ghana

Crop	Initial		Development		Mid		Late		Total
	$K_{c\text{-}ini}$	L_{ini}	$K_{c\text{-dev}}$	L_{dev}	$K_{c\text{-}mid}$	L_{mid}	$K_{c\text{-}late}$	L_{late}	
Maize	0.7	25	0.95	40	1.2	45	0.47	30	140
Rice	1.05	30	1.1	30	1.2	60	0.75	30	150
Cowpeas	0.4	20	0.73	30	1.05	30	0.47	20	110
Groundnut	0.4	25	0.77	35	1.15	45	0.6	25	130
Leafy vegie (Spinach)	0.7	20	0.85	30	1	40	0.95	10	100

References

Batjes, N.H.: Harmonized soil profile data for applications at global and continental scales: updates to the WISE database, Soil Use and Management, 25, 124-127, 2009.

FAO: Crop evapotranspiration-Guidelines for computing crop water requirements, FAO Irrigation and drainage paper 56, Food and Agriculture Organization (FAO), Rome, ISBN 92-5-104219-5, 1998.

FAO: Irrigation water management: Irrigation methods, Training manuals no 5, Food and Agriculture Organization of the United Nations (FAO), Rome, 1988.

Lu, J.B., Sun, G., McNulty, S.G., and Amatya, D.M.: A comparison of six potential evapotranspiration methods for regional use in the southeastern United States, Am. Water Resour. Assoc., 41(3), 621–633, 2005.

Panigrahi, B., and Panda, S.N.: Field test of a soil water balance simulation model, Agricultural Water Management, 58, 223-240, 2003.

Paparrizos, S., Maris, F., and Matzarakis, A.: Sensitivity analysis and comparison of various potential evapotranspiration formulae for selected Greek areas with different climate conditions, Theor. Appl. Climatol., 128, 745–759, 2017.

Rao, N. H.: Field test of a simple soil-water balance model for irrigated areas, Journal of Hydrology, 91, 179-186, 1987.

Saxton, K.E., Rawls, W.J., Romberger, J.S., and Papendick, R.I.: Estimating generalized soil-water characteristics from texture, Soil Science Society of America Journal, 50(4), 1031-1036, 1986.
